# An interpretable multimodal machine learning model integrating transvaginal ultrasound and clinical features for differentiating benign from malignant endometrial thickening in postmenopausal women

**DOI:** 10.3389/fonc.2026.1914063

**Published:** 2026-08-14

**Authors:** Ling Li, Dan Yang, Yan Zhai, Zexing Yu, Yating Wang, Xue Shi, Xin Li, Hua Li, Huiyu Ge

**Affiliations:** 1 Department of Ultrasound, Beijing Chao-yang Hospital, Capital Medical University, Beijing, China; 2 Department of Obstetrics and Gynaecology, Beijing Chao-yang Hospital, Capital Medical University, Beijing, China

**Keywords:** deep learning, endometrial neoplasms, logistic models, menopause, sensitivity and specificity, transvaginal ultrasonography

## Abstract

**Objective:**

To develop and validate an interpretable multimodal machine learning model that integrates transvaginal ultrasound (TVUS) deep learning features with clinical risk factors for differentiating benign from malignant endometrial thickening in postmenopausal women, with the aim of reducing unnecessary invasive procedures.

**Methods:**

In this retrospective single-centre diagnostic study, 601 postmenopausal women with histopathologically confirmed endometrial thickening (509 benign, 92 malignant) were identified consecutively and allocated to a training set (n = 420) and a stratified held-out test set (n = 181) at a 7:3 ratio. Deep learning features were extracted from manually segmented mid-sagittal TVUS images via four pretrained ResNet architectures and compressed into a single imaging score (DL_score) through sequential mRMR filtering and LASSO regression. Independent clinical predictors were identified by multivariate logistic regression. Ten fusion classifiers were trained on the combined feature set and evaluated by AUC, calibration, decision curve analysis, and SHAP-based interpretability.

**Results:**

In the test set, all image-based models outperformed the clinical-only model (AUC, 0.797; 95% CI, 0.707–0.888), with ResNet152 achieving the highest single-modality AUC (0.850; 95% CI, 0.782–0.917). Among the combined models, logistic regression (LR) performed best, with an AUC of 0.906 (95% CI, 0.842–0.970), an accuracy of 84.6%, a sensitivity of 75.9%, and a specificity of 92.1%. The LR model was well calibrated (Brier score, 0.08; Hosmer–Lemeshow P = 0.60) and, on DCA, provided the greatest net benefit across the clinically relevant range of threshold probabilities. SHAP analysis identified the DL_score, postmenopausal bleeding, and BMI as the three most influential predictors; Grad-CAM activation maps indicated that the DL_score captured spatially localised information related to endometrial bulk and junction integrity.

**Conclusion:**

The combined image–clinical logistic regression model demonstrated promising discriminatory and calibration performance for risk stratification of postmenopausal endometrial thickening, achieving improved specificity over conventional thickness-based criteria. This interpretable multimodal approach may serve as a useful adjunct to support clinical decision-making in identifying postmenopausal women at lower risk of malignancy, potentially reducing the burden of unnecessary invasive procedures.

## 1 Introduction

Endometrial cancer (EC) is a common gynaecological malignancy, and its global incidence rose from 15.1 per 100,000 in 1990 to 23.6 per 100,000 in 2019 (1, 2). Early diagnosis is important, but clinical triage remains difficult. Postmenopausal bleeding (PMB) is a common presenting symptom, yet only 5–15% of symptomatic women have malignancy (3). Transvaginal ultrasound (TVUS) is the first-line imaging modality; however, when an endometrial thickness cutoff of 4–5 mm is used, specificity is only 51.5% in patients with endometrial thickening (≥4 mm) (4, 5). As a result, many patients undergo invasive procedures such as hysteroscopy and diagnostic curettage that prove unnecessary.

The International Endometrial Tumour Analysis (IETA) consensus was developed to standardize evaluation, but qualitative interpretation is subject to substantial inter-observer variability (6, 7). More objective, quantitative, and reproducible methods are therefore needed. Radiomics and deep learning can extract image information that is not apparent on visual inspection (8, 9), but their application to endometrial disease has lagged behind other oncological fields (10–13). Existing machine learning studies of endometrial lesions are limited by small sample sizes, single-model designs, limited interpretability, and a lack of assessment of clinical utility (14–17).

To address these limitations, we established a modelling framework guided by three *a priori* design principles. First, multi-scale deep features were extracted from four ResNet architectures and compressed into a single imaging score through sequential mRMR(Maximum Relevance Minimum Redundancy) and LASSO-based dimensionality reduction, preventing the curse of dimensionality when fusing with clinical variables. Second, regularized logistic regression was selected as the primary classifier, as it provides stable generalization on small malignant-case cohorts and yields directly interpretable predictor weights. Third, model transparency was prioritized through SHAP attribution and Grad-CAM spatial analysis, enabling per-prediction accountability. These principles guided the design before any outcome analysis was performed. Model performance was evaluated across discrimination (AUC), calibration (Brier score, Hosmer–Lemeshow test), clinical utility (DCA), and interpretability (SHAP) to provide a comprehensive, clinically grounded assessment.

## 2 Materials and methods

### 2.1 Study design overview

**Figure 1:**
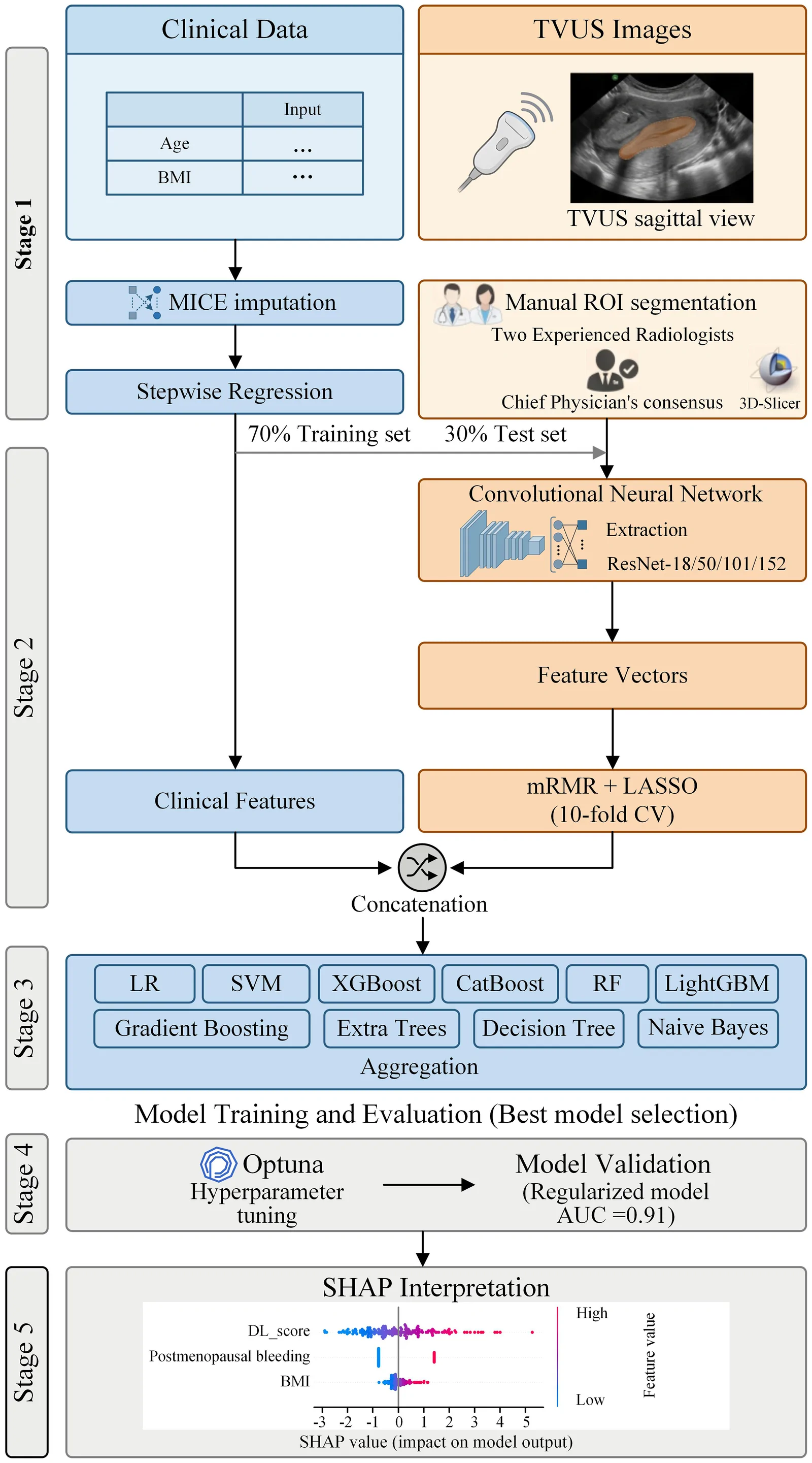
Workflow of model development and evaluation.

This study was designed, conducted, and reported in accordance with the TRIPOD guidelines for multivariable prediction model development and validation studies (18).This retrospective diagnostic accuracy study followed a four-stage analytical pipeline: (1) manual segmentation of endometrial regions of interest (ROIs) from mid-sagittal TVUS images and deep learning feature extraction via four pretrained ResNet architectures; (2) sequential dimensionality reduction of imaging features through mRMR filtering and LASSO regression, yielding a single composite imaging score (DL_score); (3) identification of independent clinical predictors via multivariate logistic regression; and (4) development and comparison of ten fusion classifiers combining the DL_score with clinical predictors, evaluated on a stratified held-out test set using AUC, calibration, decision curve analysis, and SHAP-based interpretability. All analytical decisions were made exclusively within the training set; the test set was reserved for final performance evaluation only (**Figure 1**).

### 2.2 Study population

This single-centre retrospective diagnostic study consecutively identified postmenopausal women with sonographically detected endometrial thickening who presented to our institution between January 2018 and December 2025. Inclusion criteria were: (1) natural menopause of at least 12 consecutive months; (2) endometrial thickness greater than 4 mm on standardised mid-sagittal TVUS; (3) definitive histopathological diagnosis via hysteroscopic biopsy or hysterectomy; and (4) availability of high-quality 2D grayscale TVUS images permitting complete endometrial ROI delineation. Patients were excluded if they had poor image quality precluding ROI demarcation, missing data in three or more key clinical covariates, prior or concurrent malignancies other than endometrial carcinoma (including cervical, ovarian, fallopian tube, breast, or colorectal cancer, and placental site trophoblastic tumour), previous endometrial ablation, or a history of pelvic radiotherapy or chemotherapy.

**Figure 2:**
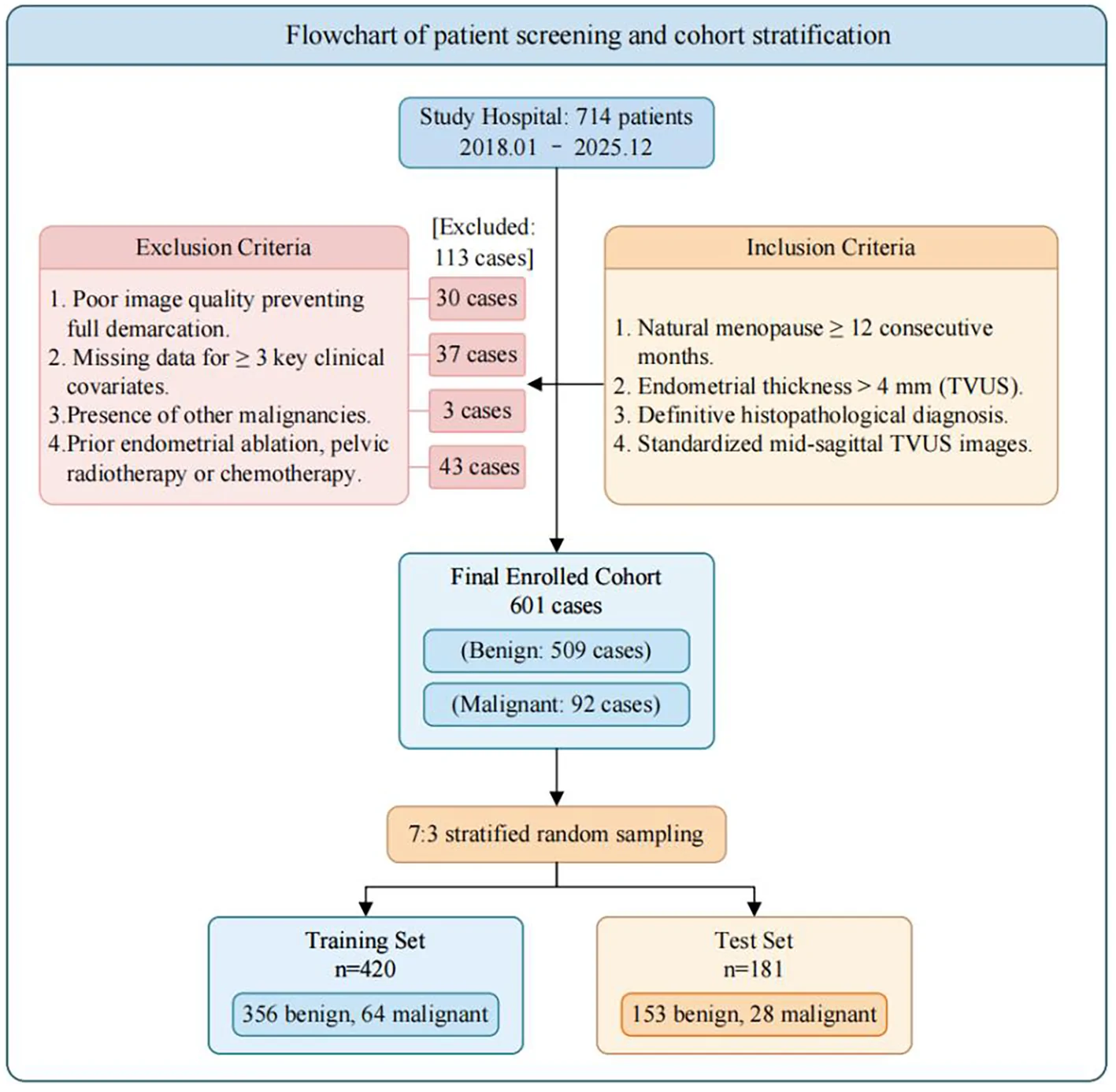
Flowchart of patient screening and cohort stratification.

Of 714 patients initially screened, 113 were excluded, leaving 601 for analysis (**Figure 2**). Of these, 509 (84.7%) had benign lesions—endometrial polyps, non-atypical hyperplasia, or submucous fibroids—and 92 (15.3%) had malignant lesions comprising atypical endometrial hyperplasia and endometrial carcinoma. The cohort was divided into a training set (n = 420; 356 benign, 64 malignant) and an independent held-out test set (n = 181; 153 benign, 28 malignant) by stratified random sampling at a 7:3 ratio, preserving the benign-to-malignant proportion across subsets.

#### 2.2.1 Sample size considerations

Consecutive eligible patients were retrospectively identified; no prospective power calculation was performed, as sample size was constrained by clinical data availability. Sample adequacy was assessed against two pre-specified thresholds. First, using the Obuchowski method for binary diagnostic tests (two-sided α = 0.05, power = 80%, prevalence 15%), at least 24 malignant cases were required in the test set to detect an AUC of 0.85 against a null of 0.70; the held-out test set contained 28 malignant cases, meeting this criterion. Second, the events-per-variable ratio in the training set was 21.3 (64 malignant events across three final predictors), exceeding the recommended minimum of 10 for stable logistic regression estimation (19).

### 2.3 Clinical and imaging data collection

Clinical variables collected at enrolment included age, age at menopause, body mass index (BMI), gravidity, parity, history of hypertension and diabetes mellitus, presence of postmenopausal bleeding (PMB), and menopausal hormone therapy (MHT) use.

All imaging data originated from routine TVUS examinations performed in our department using four ultrasound systems: Samsung WS80A with E3-12A transvaginal probe, GE Voluson E8/E10 with RIC5-9-D and eM6C intracavitary probes, Philips EPIQ Q7 with C10-3v probe, and Mindray Resona R9s with V11-3HU vaginal probe. All scans were performed by certified sonographers with at least five years of specialised gynaecological ultrasound experience. The uterine mid-sagittal plane was used as the standard reference view, as this longitudinal section displays full endometrial thickness and the endometrial–myometrial boundary clearly. High-resolution static JPEG images centred on the endometrium were exported from the hospital PACS system for subsequent analysis.

### 2.4 Data preprocessing

#### 2.4.1 Missing data

Only BMI had missing values (11 of 601 patients, 1.8%), with data missing at random. Missing values were imputed using multiple imputation by chained equations (MICE) with five imputed datasets; auxiliary predictors in the imputation model comprised age, age at menopause, gravidity, parity, hypertension, diabetes mellitus, PMB, menopausal hormone therapy (MHT) use and pathological outcome. To prevent data leakage, the imputation model was fitted exclusively on the training set and applied unchanged to the test set; results were pooled using Rubin’s rules (20).

#### 2.4.2 Class imbalance

The cohort was class-imbalanced, with malignant lesions comprising 15.3% of cases. Two strategies were applied: for logistic regression, inverse-frequency cost-sensitive class weights were assigned (benign:malignant ratio approximately 1:5.53); for ensemble tree-based models, stratified resampling was performed within each cross-validation fold rather than on the full training set, to prevent data leakage from pre-fold oversampling. Model performance was reported using AUC, sensitivity, specificity, and decision curve analysis rather than overall accuracy, given the class imbalance.

### 2.5 ROI segmentation and quality control

Endometrial ROIs were manually delineated on mid-sagittal TVUS images using 3D Slicer (version 5.2.2; www.slicer.org). Delineation encompassed the full endometrial layer of the uterine fundus and corpus; adjacent myometrium, intrauterine fluid, and pelvic free fluid were strictly excluded. For patients with more than one eligible image, all mid-sagittal frames were segmented and the resulting feature vectors averaged at the patient level.

Two radiologists with more than eight years of specialised gynaecological ultrasound experience independently performed all delineations, blinded to histopathological findings. Discrepancies were resolved by consensus adjudication with a senior radiologist, whose determination was adopted for all subsequent analyses; fewer than 5% of cases (n < 30) required this third-party review. Because features were extracted from fixed pretrained ResNet architectures whose outputs are inherently robust to minor boundary variation, formal intraclass correlation coefficient (ICC) filtering was not applied.

### 2.6 Deep learning feature extraction

Four ResNet variants pretrained on ImageNet (ResNet18, ResNet50, ResNet101, ResNet152) were used as fixed feature extractors without fine-tuning. Input ROIs were resized to 224 × 224 pixels, converted to three-channel format, and pixel values normalised to [0, 1]. Feature vectors were taken from the global average pooling layer, yielding 512 dimensions for ResNet18 and 2048 for the remaining three architectures. Where multiple images were available per patient, image-level vectors were averaged to produce a single patient-level representation.

### 2.7 Feature selection and multimodal fusion

#### 2.7.1 Imaging feature selection

**Figure 3:**
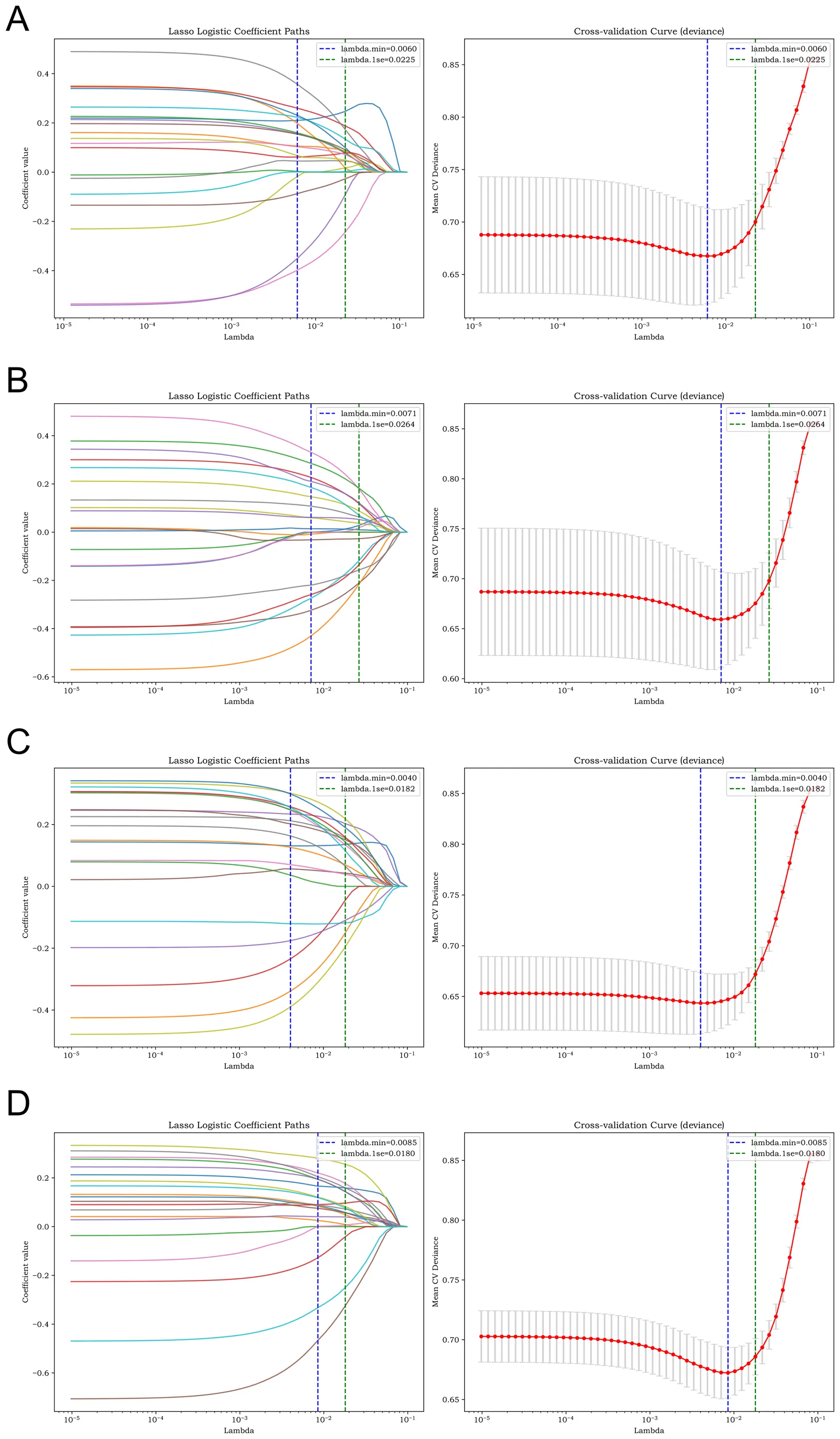
Imaging feature selection using LASSO logistic regression for the four deep learning architectures: **(A)** ResNet18, **(B)** ResNet50, **(C)** ResNet101, and **(D)** ResNet152. Left column: LASSO coefficient paths as a function of the regularization parameter (λ). Right column: 10-fold cross-validation curves used to select the optimal λ (λmin and λ1se); the mean cross-validated partial likelihood deviance is plotted against log(λ), with error bars showing the standard error. The dashed vertical lines indicate λmin (used for feature selection in this study) and λ1se (shown for reference).

Feature selection was performed in the training set only to prevent data leakage. In the first step, mRMR selected the top 20 features per ResNet architecture (80 candidates in total); the upper bound of 20 per architecture was set *a priori* to keep the candidate pool tractable given the 64 malignant training cases (21). In the second step, these 80 candidates were entered into LASSO logistic regression with the regularisation parameter λ selected by 10-fold cross-validation (λ_min criterion); features with non-zero coefficients were retained as the final imaging signature. All selection parameters were fixed before any evaluation on the test set (**Figure 3**).

#### 2.7.2 Clinical feature selection

Candidate clinical variables were screened by univariate logistic regression in the training set; those with P < 0.10 were entered into a multivariate logistic regression model with backward stepwise elimination (removal threshold P > 0.05). The surviving variables constituted the final clinical feature set.

#### 2.7.3 Late-stage fusion and DL_score derivation

A late fusion strategy was adopted, whereby each modality underwent independent feature extraction and dimensionality reduction before integration at the decision level. The 32 retained imaging features were linearly combined via their LASSO-estimated coefficients into a single scalar — the deep learning score (DL_score) — defined as the log-odds output of the LASSO logistic regression model: DL_score = β_0_ + Σβ_j_x_j_. Coefficients were estimated on the training set and applied without modification to the test set. The DL_score was then concatenated with the two independent clinical predictors (PMB and BMI) to form a three-dimensional multimodal feature vector, which served as the sole input to the ten downstream classifiers.

### 2.8 Model development and evaluation

Ten classifiers were trained on the multimodal feature vector: logistic regression (LR), support vector machine (SVM), XGBoost, random forest (RF), LightGBM, CatBoost, gradient boosting, extra trees, decision tree (DT), and naïve Bayes. Continuous features were standardised by Z-score normalisation. Hyperparameters were optimised using the Optuna framework with 10-fold cross-validation within the training set (22); parameters were fixed before evaluation on the held-out test set.

Model performance was assessed across three dimensions. Discrimination was quantified by AUC with 95% confidence intervals; sensitivity, specificity, and accuracy were reported at the Youden-index optimal threshold; pairwise AUC differences were tested using the DeLong test. Calibration was assessed by the Brier score and Hosmer–Lemeshow goodness-of-fit test, and visualised using calibration curves. Clinical utility was evaluated by decision curve analysis (DCA) across the clinically relevant range of threshold probabilities.

To quantify optimism from the full modelling pipeline, bootstrap internal validation was performed with 1,000 resamples. In each resample, the complete workflow—mRMR selection, LASSO-based DL_score derivation, clinical variable screening, and final LR fitting—was replicated. Optimism was defined as the difference between bootstrap-apparent and bootstrap-test performance; optimism-corrected estimates were obtained by subtracting the mean optimism from apparent training-set performance.

### 2.9 Model interpretability

Global and individual-level model interpretability was assessed using SHapley Additive exPlanations (SHAP); summary (beeswarm) and force plots were generated to quantify the direction and magnitude of each predictor’s contribution to malignancy risk estimates (23).

To examine the image-level basis of the DL_score, gradient-weighted class activation mapping (Grad-CAM) was applied to the terminal convolutional layer of ResNet152. Activation maps were computed from segmented endometrial ROIs following the same preprocessing pipeline used during training, then superimposed on the source TVUS images within manually annotated endometrial boundaries. Representative benign and malignant cases from the test set were selected to illustrate differences in spatial activation patterns.

## 3 Results

### 3.1 Patient baseline characteristics

A total of 601 patients with postmenopausal endometrial thickening were enrolled (509 benign and 92 malignant). Using stratified random sampling, patients were allocated to a training set (n = 420) and an independent test set (n = 181) at a 7:3 ratio. There were no significant differences between the two sets in age, age at menopause, BMI, gravidity, parity, hypertension, diabetes, or postmenopausal bleeding (all P > 0.05; **Supplementary Table 1**). The ratio of benign to malignant cases was similar in the two sets.

### 3.2 Clinical features selected by univariate screening

To identify independent clinical predictors of malignancy, univariate logistic regression was first performed on eight clinical variables (**Table 1**). Five variables were associated with malignancy at P < 0.10: age (OR, 1.03; 95% CI, 1.00–1.07; P = 0.0618), age at menopause (OR, 1.06; 95% CI, 1.00–1.12; P = 0.0498), BMI (OR, 1.18; 95% CI, 1.10–1.27; P < 0.001), gravidity (OR, 0.76; 95% CI, 0.59–0.98; P = 0.0312), and postmenopausal bleeding (OR, 6.91; 95% CI, 3.86–12.36; P < 0.001). These five variables were entered into the multivariate analysis.

**Table 1:** Univariate logistic regression analysis of clinical characteristics associated with endometrial malignancy.

Variables	Benign (n=356)	Malignant (n=64)	OR	95% CI	P-value
Age (years), median (IQR)	60.0 (55.0–66.0)	63.0 (58.0–67.0)	1.03	1.00 – 1.07	0.062
Age of menopause (years), median (IQR)	51.0 (49.0– 53.0)	51.0 (50.0–54.0)	1.06	1.00 – 1.12	0.050
BMI (kg/m²), median (IQR)	25.0 (23.1– 26.8)	27.55(24.88–30.0)	1.18	1.10 – 1.27	<0.001
Gravidity, median (IQR)	2.0 (2.0– 3.0)	2.0 (1.0–3.0)	0.76	0.59 – 0.98	0.031
Hypertension, n (%)					0.398
No	185 (52.0%)	29 (45.3%)	1	—	—
Yes	171 (48.0%)	35 (54.7%)	1.31	0.77 – 2.23	0.328
Diabetes, n (%)					0.954
No	229 (64.3%)	42 (65.6%)	1	—	—
Yes	127 (35.7%)	22 (34.4%)	0.94	0.54 – 1.65	0.842
Postmenopausal bleeding, n (%)					<0.001
No	270 (75.8%)	20 (31.2%)	1	—	—
Yes	86 (24.2%)	44 (68.8%)	6.91	3.86 – 12.36	<0.001
Parity, n (%)					0.550
0	13 (3.7%)	4 (6.2%)	1	—	—
1	267 (75.0%)	45 (70.3%)	0.55	0.17 – 1.75	0.311
2	51 (14.3%)	12 (18.8%)	0.76	0.21 – 2.76	0.682
MHT use					
No	345(96.9%)	60(93.8%)	1	—	—
Yes	11(3.1%)	4(6.3%)	2.09	0.64-6.78	0.219

Continuous variables are presented as median (IQR) and were compared using the Mann–Whitney U test. Categorical variables are presented as n (%) and were compared using the chi-square test. BMI, body mass index; CI, confidence interval; IQR, interquartile range; OR, odds ratio.

The remaining variables — including hypertension, diabetes, parity, and MHT use — did not reach significance on univariate analysis (all P > 0.05; **Table 1**) and were not considered for multivariate modelling.MHT use was recorded in 23 of 601 participants (3.8%); it did not reach significance on univariate analysis (OR 2.09, 95% CI 0.64–6.78; P = 0.219) and was not entered into multivariate modelling. No participant reported use of tamoxifen, selective oestrogen receptor modulators, progestin-only agents, tibolone, or aromatase inhibitors.

### 3.3 Clinical features selected by multivariate analysis

The five variables with P < 0.10 in the univariate analysis were entered into a multivariate logistic regression model, and independent predictors were identified using backward stepwise elimination. Postmenopausal bleeding (OR, 6.15; 95% CI, 3.60–12.14; P < 0.001) and BMI (OR, 1.18; 95% CI, 1.07–1.25; P < 0.001) were the only independent predictors of malignancy. These two variables formed the final clinical feature set used for fusion modelling (**Table 2**).

**Table 2:** Multivariate logistic regression analysis of independent clinical predictors.

Variables	OR	95% CI	P-value
Postmenopausal bleeding	6.15	3.60 - 12.14	< 0.001
BMI (kg/m²)	1.18	1.07 - 1.25	< 0.001

Multivariate logistic regression with backward stepwise elimination was used to identify independent predictors of malignancy. Variables with P < 0.10 in the univariate analysis were entered into the multivariate model. CI, confidence interval; OR, odds ratio.

### 3.4 Deep learning imaging features selected

After mRMR filtering, each of the four ResNet architectures contributed 20 features, yielding a pooled candidate set of 80 features. LASSO logistic regression with 10-fold cross-validation was then applied to this combined 80-feature pool, and 32 features with non-zero coefficients were retained as the final imaging signature based on the λmin criterion.

### 3.5 Model performance

#### 3.5.1 Comparison of ResNet architectures of different depths

To compare the ResNet variants, logistic regression was used as a standardized classifier for the deep features from ResNet18, ResNet50, ResNet101, and ResNet152 in both the training and test sets.

**Figure 4:**
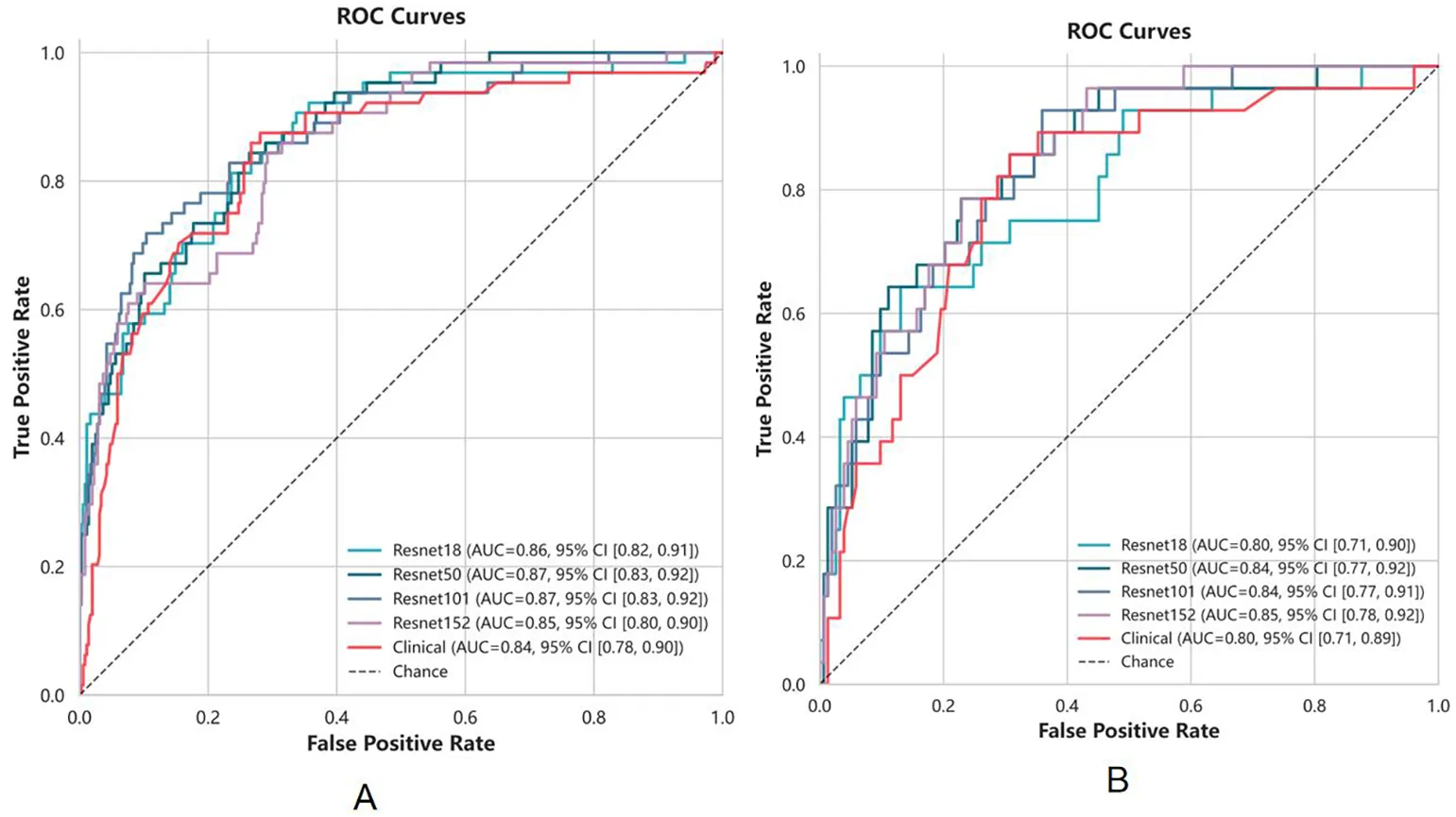
Receiver operating characteristic (ROC) curves of the four ResNet models and the clinical model in the **(A)** training set (n = 420) and **(B)** independent test set (n = 181). The clinical model includes postmenopausal bleeding and body mass index. The diagonal dashed line indicates an AUC of 0.50. Exact values and DeLong test comparisons are given in **Tables 3** and **4**.

All image-based models showed stable discrimination in both sets (**Table 3**; **Figure 4**). In the training set, AUCs ranged from 0.854 to 0.875. In the test set, ResNet152 had the highest AUC (0.850; 95% CI, 0.782–0.917), with an accuracy of 0.862 and a specificity of 0.935. The test-set AUCs for ResNet50, ResNet101, and ResNet18 were 0.844, 0.840, and 0.805, respectively. Although all image-based models had higher AUCs than the clinical-only model (test AUC, 0.797; 95% CI, 0.707–0.888), the pairwise differences were not statistically significant on the DeLong test (all P > 0.05; Section 3.4.2). This is likely related to the limited number of malignant cases (n = 92), which reduces the statistical power of the DeLong test.

**Table 3:** Diagnostic performance of the models in the training and test sets.

Model	AUC (95% CI)	Accuracy	Sensitivity	Specificity
Test Set				
ResNet18	0.805 (0.712–0.897)	0.845	0.500	0.908
ResNet50	0.844 (0.768–0.920)	0.851	0.607	0.895
ResNet101	0.840 (0.768–0.911)	0.834	0.536	0.889
ResNet152	0.850 (0.782–0.917)	0.862	0.464	0.935
Clinical	0.797 (0.707–0.888)	0.807	0.393	0.882
Training Set				
ResNet18	0.865 (0.815–0.914)	0.874	0.563	0.930
ResNet50	0.874 (0.831–0.917)	0.857	0.656	0.893
ResNet101	0.875 (0.825–0.924)	0.879	0.688	0.913
ResNet152	0.854 (0.804–0.904)	0.886	0.578	0.941
Clinical	0.841 (0.785–0.898)	0.864	0.563	0.919

Logistic regression was used as the standardized classifier. The clinical model includes the independent clinical predictors (postmenopausal bleeding and BMI). AUC, area under the receiver operating characteristic curve; CI, confidence interval.

The DeLong test showed that the pairwise AUC differences between models were not significant in either the training or the test set (all P > 0.05; **Table 4**). Although the differences between architectures did not reach statistical significance given the current number of malignant cases, the differences in clinical utility are shown by the decision curve analysis below.

**Table 4:** Pairwise comparison of clinical and ResNet-based models in the training and test sets (DeLong test).

Dataset	Model 1	Model 2	AUC1	AUC2	Z statistic	P-value	95% CI for AUC difference
Training Set	ResNet18	ResNet50	0.865	0.874	-0.415	0.678	(-0.051, 0.033)
	ResNet18	ResNet101	0.865	0.875	-0.439	0.661	(-0.054, 0.034)
	ResNet18	ResNet152	0.865	0.854	0.434	0.664	(-0.040, 0.062)
	ResNet18	Clinical	0.865	0.841	0.648	0.517	(-0.048, 0.095)
	ResNet50	ResNet101	0.874	0.875	-0.036	0.972	(-0.044, 0.043)
	ResNet50	ResNet152	0.874	0.854	0.902	0.367	(-0.024, 0.064)
	ResNet50	Clinical	0.874	0.841	0.924	0.355	(-0.037, 0.102)
	ResNet101	ResNet152	0.875	0.854	0.803	0.422	(-0.030, 0.072)
	ResNet101	Clinical	0.875	0.841	0.899	0.369	(-0.039, 0.106)
	ResNet152	Clinical	0.854	0.841	0.337	0.736	(-0.060, 0.084)
Test Set	ResNet18	ResNet50	0.805	0.844	-1.241	0.215	(-0.102, 0.023)
	ResNet18	ResNet101	0.805	0.840	-0.916	0.359	(-0.109, 0.040)
	ResNet18	ResNet152	0.805	0.850	-1.242	0.214	(-0.116, 0.026)
	ResNet18	Clinical	0.805	0.797	0.159	0.874	(-0.088, 0.103)
	ResNet50	ResNet101	0.844	0.840	0.159	0.874	(-0.053, 0.062)
	ResNet50	ResNet152	0.844	0.850	-0.171	0.864	(-0.067, 0.056)
	ResNet50	Clinical	0.844	0.797	1.019	0.308	(-0.043, 0.138)
	ResNet101	ResNet152	0.840	0.850	-0.282	0.778	(-0.080, 0.060)
	ResNet101	Clinical	0.840	0.797	0.891	0.373	(-0.051, 0.136)
	ResNet152	Clinical	0.850	0.797	0.995	0.320	(-0.051, 0.156)

The DeLong test was used to compare AUCs between models in the training and test sets. P < 0.05 indicates a significant difference. AUC, area under the receiver operating characteristic curve; CI, confidence interval.

**Figure 5:**
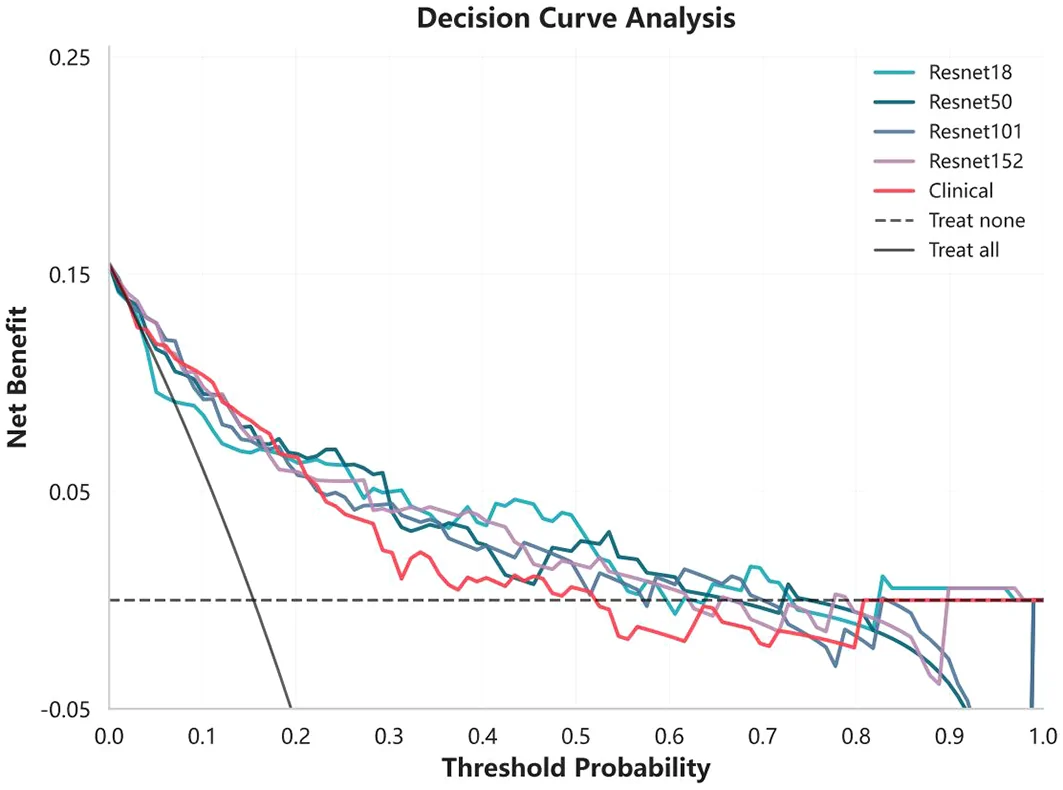
Decision curve analysis of the clinical model and the four ResNet models. Across the threshold probability range of 10–50%, the combined models provided higher net benefit than the “treat all” and “treat none” strategies and than the clinical model alone.

Decision curve analysis was used to compare the clinical utility of the models (**Figure 5**). All four image-based ResNet models provided higher net benefit than the clinical-only model across a threshold probability range of 0.10 to 0.50, and ResNet152 had the highest net benefit across most of this range. For example, at a threshold probability of 0.30—a value commonly used in gynaecological practice to decide whether to perform endometrial biopsy—the net benefit was approximately 0.065 for ResNet152, compared with approximately 0.035 for the clinical model and approximately 0.050 for ResNet18. These findings indicate that adding deep features from the deeper networks increased net benefit relative to clinical variables alone.

#### 3.5.2 Comparison of machine learning algorithms

**Figure 6:**
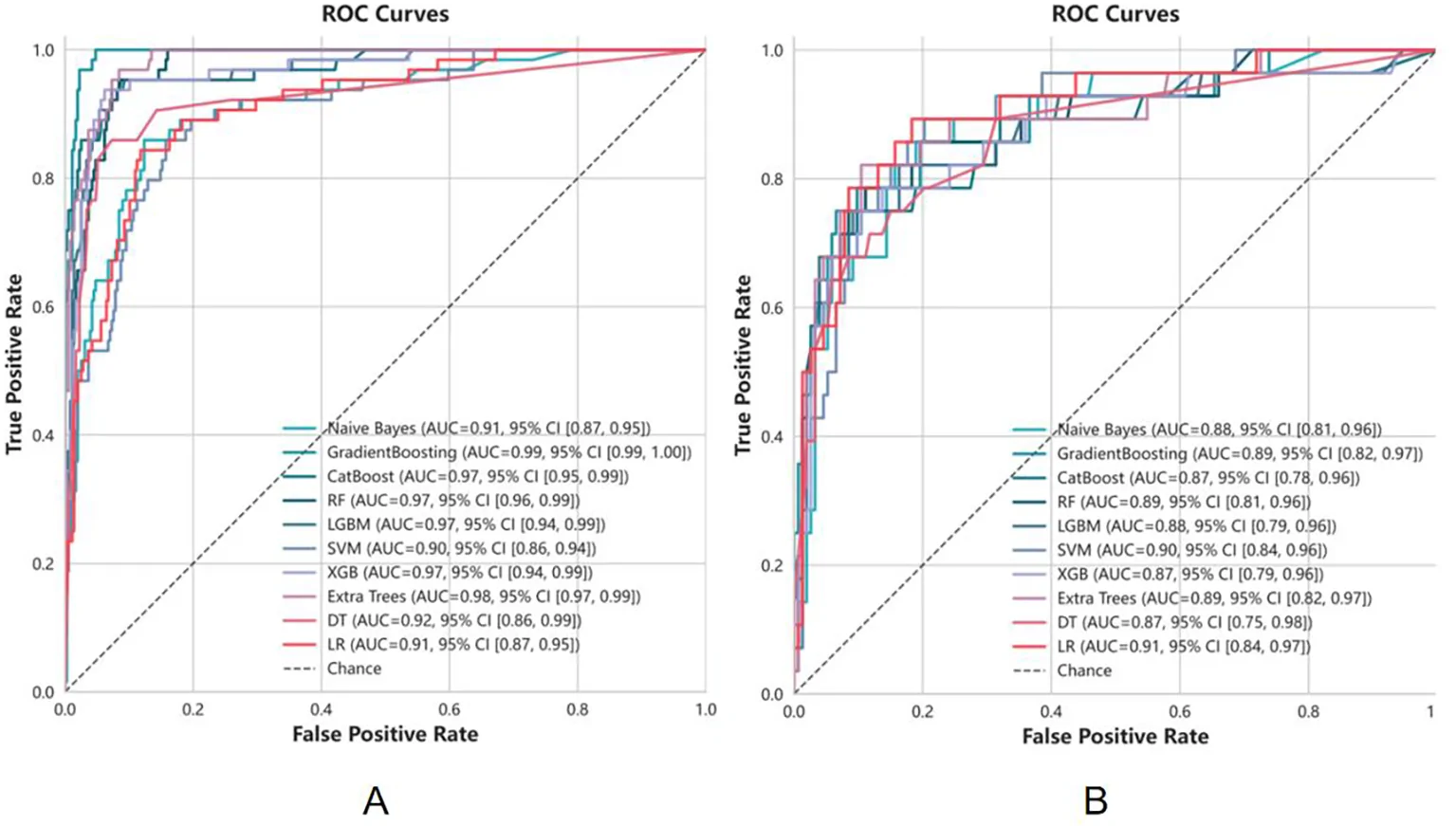
ROC curves of the ten machine learning models in the **(A)** training set and **(B)** test set. The diagonal dashed line indicates an AUC of 0.50; AUC values and 95% CIs are shown in the image. DT, decision tree; LR, logistic regression; RF, random forest; SVM, support vector machine; XGB, XGBoost.

Using the combined image–clinical feature set, the ten machine learning algorithms were compared (**Figure 6**). In the training set, the ensemble models—gradient boosting (GB), random forest (RF), LightGBM (LGBM), and XGBoost (XGB)—had high AUCs (0.97 to 0.99), whereas logistic regression (LR) and support vector machine (SVM) had lower training AUCs of 0.906 and 0.900, respectively.

The AUCs of the ensemble models on the test set ranged from 0.866 to 0.893, compared with training AUCs of 0.97–0.99, consistent with overfitting. In contrast, the LR model maintained a test AUC of 0.906 (95% CI, 0.84–0.97), followed by SVM (AUC, 0.897; 95% CI, 0.84–0.96).

On the DeLong test, LR achieved a significantly higher AUC than Naive Bayes (0.884; Z = 2.162; P = 0.031), CatBoost (0.866; Z = 2.022; P = 0.043), and XGBoost (0.871; Z = 2.015; P = 0.044). No significant differences were observed between LR and SVM (0.897; P = 0.379), Gradient Boosting (0.893; P = 0.382), RF (0.888; P = 0.217), LightGBM (0.878; P = 0.101), Extra Trees (0.893; P = 0.444), or DT (0.868; P = 0.249) (**Table 5**).

**Table 5:** Pairwise comparison of AUC differences between the LR model and all other machine learning classifiers on the independent test set (DeLong’s test).

Model pair	AUC_1_(LR)	AUC_2_	Z statistic	P-value	95% CI
LR vs Naive Bayes	0.906	0.884	2.162	0.031★	(0.002, 0.042)
LR vs CatBoost	0.906	0.866	2.022	0.043★	(0.001, 0.079)
LR vs XGBoost	0.906	0.871	2.015	0.044★	(0.001, 0.069)
LR vs LightGBM	0.906	0.878	1.640	0.101	(−0.006, 0.063)
LR vs RF	0.906	0.888	1.236	0.217	(−0.011, 0.047)
LR vs DT	0.906	0.868	1.153	0.249	(−0.027, 0.105)
LR vs SVM	0.906	0.897	0.879	0.379	(−0.011, 0.030)
LR vs Gradient Boosting	0.906	0.893	0.874	0.382	(−0.017, 0.045)
LR vs Extra Trees	0.906	0.893	0.766	0.444	(−0.021, 0.048)

AUC₁, area under the ROC curve for logistic regression (LR); AUC₂, AUC for the comparator model; Z, (AUC₁ − AUC₂)/SE, where SE was estimated using the DeLong method; 95% CI, 95% confidence interval for the AUC difference (AUC₁ − AUC₂); positive values favour LR. All tests were two-sided; statistical significance was defined as P < 0.05. Models are ranked in ascending order of P-value. ^★^P < 0.05. AUC, area under the receiver operating characteristic curve; CI, confidence interval; DT, decision tree; LR, logistic regression; RF, random forest; SVM, support vector machine.

#### 3.5.3 Model calibration

**Figure 7:**
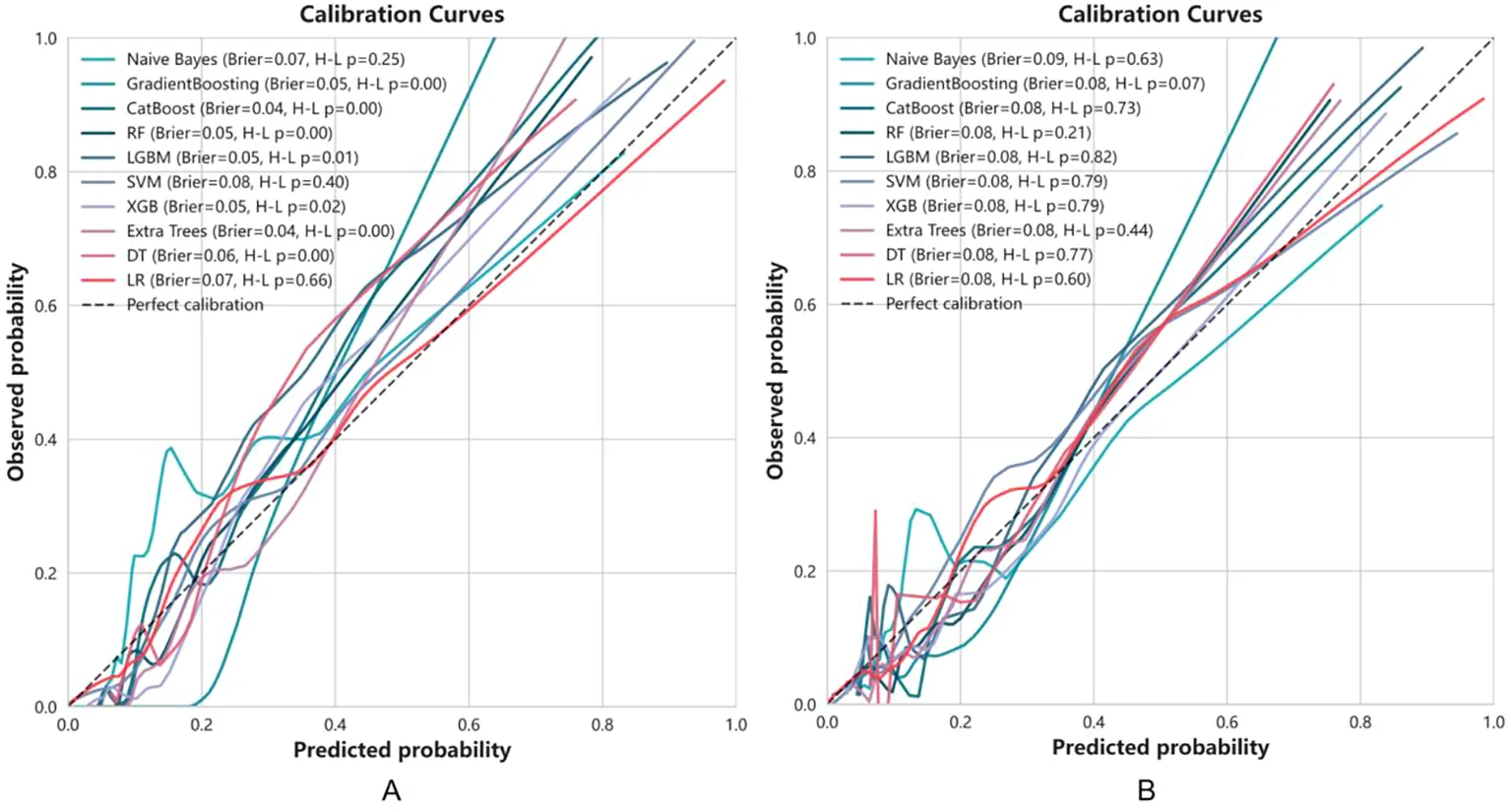
Calibration curves of the machine learning models in the **(A)** training set and **(B)** test set. The x-axis shows the predicted probability of malignancy and the y-axis the observed malignancy rate; curves closer to the diagonal indicate better calibration. Brier scores and Hosmer–Lemeshow P values are shown in the figure.

Calibration was assessed using the Brier score and the Hosmer–Lemeshow (H–L) test. In the training set, LR had the best calibration (Brier score, 0.07; H–L P = 0.66). The ensemble models, such as gradient boosting and random forest, showed poorer calibration (all H–L P < 0.05), consistent with overfitting during training (**Figure 7**).

In the test set, LR maintained good calibration (Brier score, 0.08; H–L P = 0.60), with a calibration curve close to the diagonal. The other models also calibrated reasonably well in the test set, with Brier scores between 0.08 and 0.09 and all H–L P > 0.05. These results indicate that the probabilities predicted by the LR model were reliable.

#### 3.5.4 Performance of the combined model

Based on the evaluations above, the combined image–clinical LR model was selected as the final model. In the independent test set, it had an AUC of 0.906 (95% CI, 0.84–0.97) for distinguishing benign from malignant endometrial lesions, with an accuracy of 84.6%, a sensitivity of 75.9%, and a specificity of 92.1%. The model was well calibrated and had the highest net benefit across the clinically relevant threshold range on DCA.

Bootstrap internal validation (1,000 resamples, full-pipeline replication) yielded a mean optimism of 0.057 in AUC; the optimism-corrected AUC was 0.847, the corrected Brier score was 0.138, and the corrected calibration slope was 0.715, reflecting residual optimism attributable to the limited malignant-case training sample(**Table 6**).

**Table 6:** Bootstrap-based optimism correction of the final logistic regression model (1,000 resamples).

Phase	AUC	Calibration intercept	Calibration slope	Brier score	Phase
Apparent	0.904	0.000	1.000	0.104	Apparent
Optimism-corrected	0.847	-0.213	0.715	0.138	Optimism-corrected

Calibration and discrimination metrics were estimated using 1,000 full-pipeline patient-level bootstrap resamples.

### 3.6 Model interpretability

**Figure 8:**
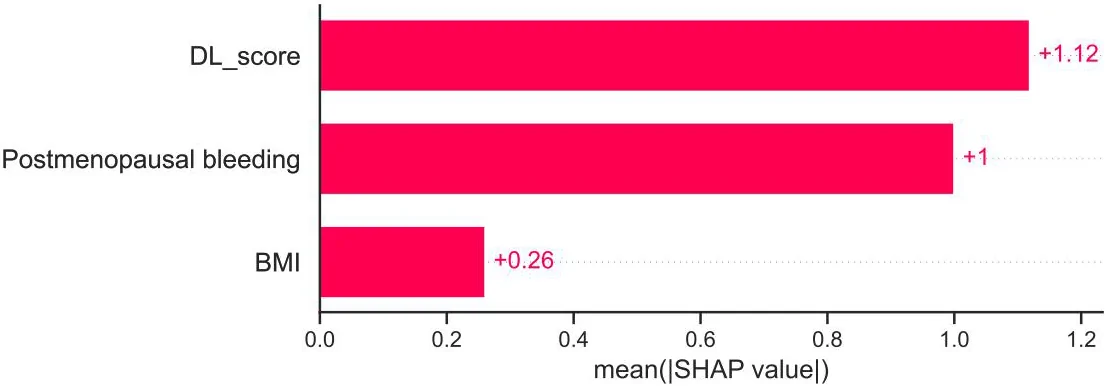
Global feature importance of the combined image–clinical model, ranked by mean |SHAP value|. The deep learning score (DL_score) had the highest importance, followed by postmenopausal bleeding and body mass index.

**Figure 9:**
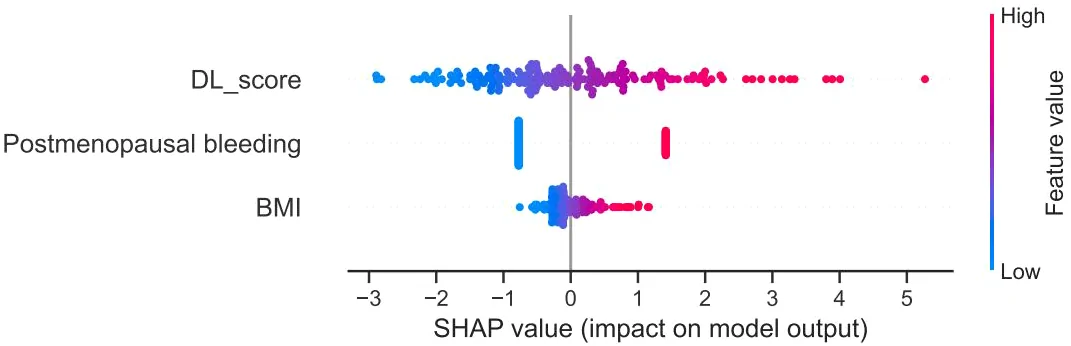
SHAP summary (beeswarm) plot for the top features of the combined image–clinical model. Each dot is a patient in the test set; higher feature values (to the right of zero) indicate a higher contribution to predicted malignancy risk. The DL_score had the widest range of SHAP values (approximately −3.0 to +5.3).

SHAP analysis of the final LR model identified the DL_score as the most influential predictor (mean |SHAP value|, 1.12), followed by postmenopausal bleeding (1.00) and BMI (0.26) (**Figure 8**). The beeswarm plot confirmed that all three predictors acted in clinically expected directions: a higher DL_score, the presence of postmenopausal bleeding, and a higher BMI each shifted individual predictions toward malignancy (**Figure 9**). The particularly wide SHAP value range for the DL_score (approximately −3.0 to +5.3) indicated that image-derived features accounted for the principal source of variation in individual risk estimates across the test set.

**Figure 10:**
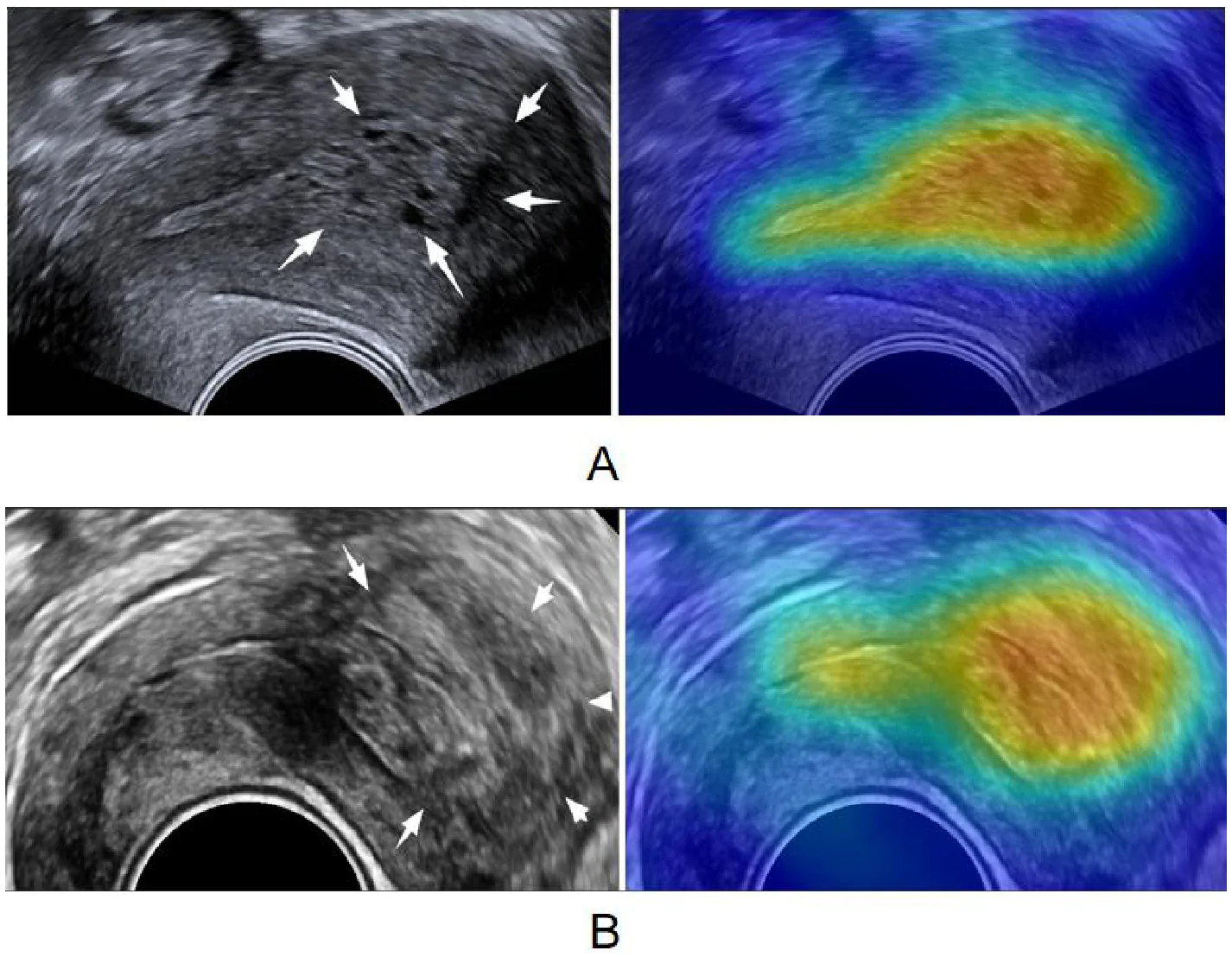
Grad-CAM activation maps generated from the ResNet152 architecture for a representative benign case **(A)** and a malignant case **(B)** of postmenopausal endometrial thickening. Activation intensity ranges from low (blue) to high (yellow/red). Activation maps were computed on segmented endometrial ROI images and superimposed on the source TVUS images for anatomical reference.

To investigate the image-level basis of the DL_score, Grad-CAM was applied to the ResNet152 architecture, and the resulting activation maps were projected onto the original TVUS images within the segmented endometrial boundary (**Figure 10**). The two representative cases demonstrated clearly divergent activation patterns. In the benign case (**Figure 10A**), high-activation regions were confined within the lesion body and attenuated rapidly toward the periphery, without focal extension toward the endometrial–myometrial junction. In the malignant case (**Figure 10B**), the spatial extent of high-activation regions was substantially greater, consistent with the marked endometrial thickening that characterizes malignant lesions. More specifically, the outer margin of the activation zone was irregular and non-contiguous, extending peripherally toward the endometrial–myometrial junction with focal protrusion into the adjacent myometrium at the lateral border—spatially corresponding to the sonographically disrupted junction integrity identified in this case. These activation patterns suggest that the DL_score encodes quantitative information pertaining to endometrial bulk and the structural integrity of the endometrial–myometrial junction.

## 4 Discussion

The principal finding of this study is that multimodal fusion of quantitative TVUS deep learning features with two independent clinical predictors—postmenopausal bleeding and BMI—yielded a combined model that outperformed either modality alone and achieved the highest discrimination among all tested configurations. Crucially, this performance advantage was delivered by a regularised three-variable logistic regression rather than by complex ensemble methods, reinforcing a principle increasingly recognised in small-malignancy-case prediction tasks: when the number of outcome events is limited, parsimony and regularisation outperform capacity. SHAP attribution further established that the imaging score was the dominant predictor, with PMB and BMI each contributing independently—a pattern that mirrors the clinical logic of risk stratification in postmenopausal endometrial disease.

Among the image-only models, the deeper ResNet152 architecture achieved the highest discriminatory and clinical utility performance, although pairwise AUC differences across architectures did not reach statistical significance, likely reflecting the limited number of malignant cases available for testing. Deeper networks have larger receptive fields and a greater capacity to model non-linear relationships (24), which can help capture image features—such as echo heterogeneity and irregularity of the endometrial–myometrial border—that are difficult to assess visually (25).In this study, the advantage of deeper networks was more apparent in clinical utility than in overall discrimination: ResNet152 had the highest net benefit on DCA, supporting its potential value for risk stratification and triage.

A notable finding was the stability of the simpler classifiers in the multimodal model. The ensemble models (e.g., gradient boosting, random forest) performed almost perfectly in training (AUC, 0.97–0.99) but showed greater AUC shrinkage on the independent test set, consistent with overfitting; although these differences did not reach statistical significance on the DeLong test, the numerically superior and more stable performance of LR across discrimination, calibration, and DCA supported its selection as the final model. The LR model did not show this decline and maintained a test AUC of 0.906. Combining features from different networks after LASSO selection may introduce collinearity and reduce the events-per-variable (EPV) ratio below conventional thresholds; the regularized LR model was less prone to overfitting in this setting with a limited training sample (n = 64 malignant cases). Bootstrap-based internal validation (1,000 resamples) confirmed this interpretation: the mean AUC optimism was 0.057, yielding a corrected AUC of 0.847—a level of shrinkage consistent with modelling on 64 malignant cases and substantially lower than the optimism observed for the ensemble methods.

The SHAP results were consistent with known mechanisms in gynaecological oncology. PMB can reflect abnormal endometrial vascularization or tissue instability (26), and a higher BMI is a recognized marker of hyperestrogenism, a driver of type I endometrial carcinoma (27). The wide range of SHAP values for the DL_score (approximately −3.0 to +5.3) indicates that the quantitative ultrasound features were the main contributor to risk stratification, with the clinical variables providing additional information. The qualitative IETA scoring system reported by Dueholm et al. (28) prioritized sensitivity (92%, with 84% specificity) in a selected, symptomatic cohort, whereas our model favoured specificity (92.1%, with 75.9% sensitivity) in a broader population. A higher-specificity model reduces false-positive results and may spare lower-risk women unnecessary diagnostic biopsies. Our model compared favourably with published ultrasound-based approaches: the radiomics models of Moro et al. (29) and Capasso et al. (30) reported validation AUCs of 0.88, and the multimodal deep learning network of Lin et al. (31) achieved 0.892 in a multicentre cohort. The present model reached 0.906 while using only three input variables, offering a more transparent decision pathway without meaningful sacrifice in discrimination.

The Grad-CAM analysis offered spatial context for interpreting the DL_score and identified two image-level features that distinguished the malignant from the benign activation pattern. First, the overall spatial extent of high-activation regions was substantially larger in the malignant case, consistent with the clinical observation that malignant lesions exhibited significantly higher endometrial thickness than benign lesions (32). Second, the activation boundary in the malignant case was irregular and extended toward the endometrial–myometrial junction, with focal protrusion into the adjacent myometrium—directly corresponding to the sonographic criterion of an irregular, interrupted endometrial–myometrial junction that is closely associated with endometrial malignancy (33). Neither feature was apparent in the benign case, where activation remained confined within the lesion body with a smooth peripheral boundary. These findings indicate that the DL_score is not a purely abstract numerical representation but encodes spatially localized information about endometrial thickness and junction integrity—two of the most diagnostically relevant, yet interobserver-variable, features in the ultrasound evaluation of postmenopausal endometrial thickening.

Several limitations of this study merit consideration. First, the retrospective single-centre design carries an inherent risk of selection bias toward more complex presentations, and verification bias cannot be excluded: biopsy referral decisions were partly informed by ultrasound findings, so patients whose imaging fell below the referral threshold were systematically unavailable for analysis. Prospective external validation in multicentre cohorts is required before the model can be considered for clinical translation. Second, the relatively small number of malignant cases (n = 92) limits subgroup analysis by histological subtype; rarer subtypes such as serous carcinoma and carcinosarcoma, which may carry distinct imaging signatures, were underrepresented. Third, manual ROI delineation is time-consuming and operator-dependent; although inter-observer discrepancies were infrequent (< 5%) and resolved by senior adjudication, development of automated segmentation would be necessary for scalable deployment. Fourth, only two-dimensional greyscale images were analysed; colour Doppler, three-dimensional ultrasound, contrast-enhanced ultrasound, and shear-wave elastography were not incorporated and may provide complementary diagnostic value (34, 35). Fifth, images were acquired across four ultrasound systems from different manufacturers, and device-stratified performance was not formally evaluated. Finally, the model outputs probabilistic risk estimates intended to complement—not replace—established clinical pathways including hysteroscopy and endometrial sampling; prospective studies with predefined decision thresholds and outcome follow-up are needed to determine its clinical utility.

## 5 Conclusion

We developed and validated a multimodal machine learning model integrating TVUS deep learning features with key clinical predictors—postmenopausal bleeding and BMI—to support non-invasive risk stratification of postmenopausal endometrial thickening. In the independent test set, the regularized LR model demonstrated satisfactory discriminatory performance, good calibration, and transparent decision attribution through SHAP analysis. These findings suggest that combining quantitative ultrasound features with clinical variables has the potential to improve specificity beyond conventional thickness-based criteria. The proposed model may provide an objective and interpretable tool to assist clinicians in identifying lower-risk patients, thereby supporting more individualized decisions regarding invasive sampling and contributing to more efficient clinical workflows.

## Data Availability

The raw data supporting the conclusions of this article will be made available by the authors, without undue reservation.

## Supplementary material

Supplementary Table 1 Baseline demographic and clinical characteristics of the study participants in the training and test sets.

## References

[1] Sung H Ferlay J Siegel RL Laversanne M Soerjomataram I Jemal A . Global cancer statistics 2020: GLOBOCAN estimates of incidence and mortality worldwide for 36 cancers in 185 countries. CA Cancer J Clin. (2021) 71:209–49. doi: 10.3322/caac.21660 33538338

[2] GBD 2019 Cancer Risk Factors Collaborators . The global burden of cancer attributable to risk factors, 2010–2019: a systematic analysis for the Global Burden of Disease Study 2019. Lancet. (2022) 400:563–91. doi: 10.1016/S0140-6736(22)01438-6 35988567 PMC9395583

[3] Clarke MA Long BJ Del Mar Morillo A Arbyn M Bakkum-Gamez JN Wentzensen N . Association of endometrial cancer risk with postmenopausal bleeding in women: a systematic review and meta-analysis. JAMA Intern Med. (2018) 178:1210–22. doi: 10.1001/jamainternmed.2018.2820 30083701 PMC6142981

[4] Concin N Matias-Guiu X Vergote I Cibula D Mirza MR Marnitz S . ESGO/ESTRO/ESP guidelines for the management of patients with endometrial carcinoma. Int J Gynecol Cancer. (2021) 31:12–39. doi: 10.1136/ijgc-2020-002230 33397713

[5] Wong AS Lao TT Cheung CW Yeung SW Fan HL Ng PS . Reappraisal of endometrial thickness for the detection of endometrial cancer in postmenopausal bleeding: a retrospective cohort study. BJOG. (2016) 123:439–46. doi: 10.1111/1471-0528.13342 25800522

[6] Leone FPG Timmerman D Bourne T Valentin L Epstein E Goldstein SR . Terms, definitions and measurements to describe the sonographic features of the endometrium and uterine cavity: a consensus opinion from the International Endometrial Tumor Analysis (IETA) group. Ultrasound Obstet Gynecol. (2010) 35:103–12. doi: 10.1002/uog.7487 20014360

[7] Green RW Epstein E IETA group . Interobserver reliability of ultrasound characteristics of the endometrium in women with postmenopausal bleeding. Ultrasound Obstet Gynecol. (2018) 52:534–40. doi: 10.1002/uog.18957 29148609

[8] Lambin P Rios-Velazquez E Leijenaar R Carvalho S van Stiphout RG Granton P . Radiomics: extracting more information from medical images using advanced feature analysis. Eur J Cancer. (2012) 48:441–6. doi: 10.1016/j.ejca.2011.11.036 22257792 PMC4533986

[9] LeCun Y Bengio Y Hinton G . Deep learning. Nature. (2015) 521:436–44. doi: 10.1038/nature14539 26017442

[10] Park VY Han K Seong YK Park MH Kim EK Moon HJ . Diagnosis of thyroid nodules: performance of a deep learning convolutional neural network model vs. radiologists. Sci Rep. (2019) 9:17843. doi: 10.1038/s41598-019-54434-1 31780753 PMC6882804

[11] Shen Y Shamout FE Oliver JR Witowski J Kannan K Park J . Artificial intelligence system reduces false-positive findings in the interpretation of breast ultrasound exams. Nat Commun. (2021) 12:5645. doi: 10.1038/s41467-021-26023-2 34561440 PMC8463596

[12] Gao Y Zeng S Xu X Li H Yao S Song K . Deep learning-enabled pelvic ultrasound images for accurate diagnosis of ovarian cancer in China: a retrospective, multicentre, diagnostic study. Lancet Digit Health. (2022) 4:e179–87. doi: 10.1016/S2589-7500(21)00278-8 35216752

[13] Hosny A Parmar C Quackenbush J Schwartz LH Aerts HJWL . Artificial intelligence in radiology. Nat Rev Cancer. (2018) 18:500–10. doi: 10.1038/s41568-018-0016-5 29777175 PMC6268174

[14] Urushibara A Saida T Mori K Ishiguro T Inoue K Masumoto T . The efficacy of deep learning models in the diagnosis of endometrial cancer using MRI: a comparison with radiologists. BMC Med Imaging. (2022) 22:80. doi: 10.1186/s12880-022-00808-3 35501705 PMC9063362

[15] Otani S Himoto Y Nishio M Fujimoto K Moribata Y Yakami M . Radiomic machine learning for pretreatment assessment of prognostic risk factors for endometrial cancer and its effects on radiologists' decisions of deep myometrial invasion. Magn Reson Imaging. (2022) 85:161–7. doi: 10.1016/j.mri.2021.10.024 34687853

[16] Liu X Qin X Luo Q Qiao J Xiao W Zhu Q . A transvaginal ultrasound-based deep learning model for the noninvasive diagnosis of myometrial invasion in patients with endometrial cancer: comparison with radiologists. Acad Radiol. (2024) 31:2818–26. doi: 10.1016/j.acra.2023.12.035 38182443

[17] Bi Q Wang Y Deng Y Liu Y Pan Y Song Y . Deep learning and radiomics in endometrial lesions: clinical application gaps and the need for interpretability. Acad Radiol. (2024) 31:1452–63. doi: 10.1016/j.acra.2023.08.012 37714718

[18] Collins GS Reitsma JB Altman DG Moons KGM . Transparent reporting of a multivariable prediction model for individual prognosis or diagnosis (TRIPOD): the TRIPOD statement. Ann Intern Med. (2015) 162:55–63. doi: 10.7326/M14-0697 25560714

[19] Riley RD Ensor J Snell KIE . Calculating the sample size required for developing a clinical prediction model. BMJ. (2020) 368:m441. doi: 10.1136/bmj.m441 32188600

[20] van Buuren S Groothuis-Oudshoorn K . mice: multivariate imputation by chained equations in R. J Stat Softw. (2011) 45:1–67. doi: 10.18637/jss.v045.i03

[21] Peng HC Long F Ding C . Feature selection based on mutual information: criteria of max-dependency, max-relevance, and min-redundancy. IEEE Trans Pattern Anal Mach Intell. (2005) 27:1226–38. doi: 10.1109/TPAMI.2005.159 16119262

[22] Akiba T Sano S Yanase T Ohta T Koyama M . Optuna: a next-generation hyperparameter optimization framework. In: Proceedings of the 25th ACM SIGKDD International Conference on Knowledge Discovery and Data Mining (KDD '19). ACM (2019) 2623–31. doi: 10.1145/3292500.3330701

[23] Lundberg SM Lee SI . A unified approach to interpreting model predictions. In: Advances in Neural Information Processing Systems 30 (NeurIPS 2017). Curran Associates (2017) 4765–74. Available online at: https://proceedings.neurips.cc/paper_files/paper/2017/file/8a20a8621978632d76c43dfd28b67767-Paper.pdf (Accessed July 15, 2026).

[24] Zhou SK Greenspan H Davatzikos C Duncan JS Van Ginneken B Madabhushi A . A review of deep learning in medical imaging: imaging traits, technology trends, case studies with progress highlights, and future promises. Proc IEEE. (2021) 109:820–38. doi: 10.1109/JPROC.2021.3054390 37786449 PMC10544772

[25] Chen X Wang X Zhang K Fung KM Thai TC Moore K . Recent advances and clinical applications of deep learning in medical image analysis. Med Image Anal. (2022) 79:102444. doi: 10.1016/j.media.2022.102444 35472844 PMC9156578

[26] Middelkoop MA Don EE Hehenkamp WJK Polman NJ Griffioen AW Huirne JAF . Angiogenesis in abnormal uterine bleeding: a narrative review. Hum Reprod Update. (2023) 29:457–85. doi: 10.1093/humupd/dmad004 36857162 PMC10320491

[27] Kitson S Ryan N MacKintosh ML Edmondson R Duffy JMN Crosbie EJ . Interventions for weight loss in obesity to reduce endometrial cancer risk: a Cochrane systematic review. Cochrane Database Syst Rev. (2021) 2:CD012513. doi: 10.1002/14651858.CD012513.pub2 29388687 PMC6491136

[28] Dueholm M Hjorth IMD Dahl K Pedersen LK Ørtoft G . Identification of endometrial cancers and atypical hyperplasia: Development and validation of a simplified system for ultrasound scoring of endometrial pattern. Maturitas. (2019) 123:15–24. doi: 10.1016/j.maturitas.2019.01.011 31027672

[29] Moro F Albanese M Boldrini L Chiappa V Lenkowicz J Bertolina F . Developing and validating ultrasound-based radiomics models for predicting high-risk endometrial cancer. Ultrasound Obstet Gynecol. (2022) 60:256–68. doi: 10.1002/uog.24805 34714568

[30] Capasso I Cucinella G Wright DE Takahashi H De Vitis LA Gregory AV . Artificial intelligence model for enhancing the accuracy of transvaginal ultrasound in detecting endometrial cancer and endometrial atypical hyperplasia. Int J Gynecol Cancer. (2024) 34:1547–55. doi: 10.1136/ijgc-2024-005652 39089731

[31] Lin C Chen W Lai J Huang J Ye X Chen S . Integrating deep learning and clinical characteristics for early prediction of endometrial cancer using multimodal ultrasound imaging: a multicenter study. Front Oncol. (2025) 15:1600242. doi: 10.3389/fonc.2025.1600242 40697376 PMC12279480

[32] Thoprasert P Phaliwong P Smanchat B Prommas S Bhamarapravatana K Suwannarurk K . Endometrial thickness measurement as predictor of endometrial hyperplasia and cancer in perimenopausal uterine bleeding: cross-sectional study. Asian Pac J Cancer Prev. (2023) 24:693–9. doi: 10.31557/APJCP.2023.24.2.693 36853321 PMC10162612

[33] Epstein E Fischerova D Valentin L Testa AC Franchi D Sladkevicius P . Ultrasound characteristics of endometrial cancer as defined by International Endometrial Tumor Analysis (IETA) consensus nomenclature. Ultrasound Obstet Gynecol. (2018) 51:818–28. doi: 10.1002/uog.18739 28944985

[34] Green RW Epstein E . Dynamic contrast-enhanced ultrasound improves diagnostic performance in endometrial cancer staging. Ultrasound Obstet Gynecol. (2020) 56:96–105. doi: 10.1002/uog.21885 31647145

[35] Guler AH Ates MC Avcı F Seher N Cintesun E Bilgi A . The role of shear wave elastography in predicting endometrial cancer in patients presenting with abnormal uterine bleeding. Eur Rev Med Pharmacol Sci. (2024) 28:365–72. doi: 10.26355/eurrev_202401_35055 38235888

