# Supplementary material for: An interpretable multimodal machine learning model integrating transvaginal ultrasound and clinical features for differentiating benign from malignant endometrial thickening in postmenopausal women
Source: Front Oncol. 2026 Aug 14;16:1914063. doi: 10.3389/fonc.2026.1914063 (PMC13521913; doi:10.3389/fonc.2026.1914063)
Supplement: Supplementary file 1 [file DataSheet1.pdf]

Supplementary Table 1. Baseline demographic and clinical characteristics of the study participants in the training and test sets.

Variables	Training set (n=420)	Test set (n=181)	P-value
Pathological Diagnosis, n (%)			0.963
Benign	356 (84.8%)	153 (84.5%)	
Malignant	64 (15.2%)	28 (15.5%)	
Age (years), median (IQR)	61.0 (55.0–67.0)	61.0 (56.0–66.0)	0.750
Age of menopause (years), median (IQR)	51.0 (50.0–53.0)	51.0 (49.0–53.0)	0.888
Postmenopausal bleeding, n (%)			0.873
No	276 (65.7%)	117 (64.6%)	
Yes	144 (34.3%)	64 (35.4%)	
BMI (kg/m²), median (IQR)	25.4 (23.5–27.3)	25.2 (23.5–28.7)	0.275
Hypertension, n (%)			0.192
No	214 (51.0%)	81 (44.8%)	
Yes	206 (49.0%)	100 (55.2%)	
Diabetes mellitus, n (%)			0.539
No	268 (63.8%)	110 (60.8%)	
Yes	152 (36.2%)	71 (39.2%)	
Gravidity, median (IQR)	2.0 (1.0–3.0)	2.0 (1.0–3.0)	0.387
Parity, n (%)			0.667
0	19 (4.5%)	7 (3.9%)	
1	310 (73.8%)	137 (75.7%)	
2	69 (16.4%)	25 (13.8%)	
≥3	22 (5.2%)	12 (6.7%)	
MHT use, n (%)			0.539
No	405 (96.43%)	173(95.6%)	
Yes	15(3.57%)	8 (4.4%)	

Note: Continuous variables are presented as median (IQR) and were compared using the Mann–Whitney U test. Categorical variables are presented as n (%) and were compared using the chi-square test. BMI, body mass index; IQR, interquartile range.
